# Elevated serum levels of soluble CD154 in children with juvenile idiopathic arthritis

**DOI:** 10.1186/1546-0096-6-8

**Published:** 2008-05-28

**Authors:** Sampath Prahalad, Thomas B Martins, Anne E Tebo, April Whiting, Bronte Clifford, Andrew S Zeft, Bernadette McNally, John F Bohnsack, Harry R Hill

**Affiliations:** 1 Department of Pediatrics, University of Utah, Salt Lake City, UT, USA; 2 The Associated Regional and University Pathologists Institute for Clinical and Experimental Pathology, Salt Lake City, UT, USA; 3 Department of Pathology, University of Utah, Salt Lake City, UT, USA; 4 Department of Medicine, University of Utah, Salt Lake City, UT, USA

## Abstract

**Objective:**

Cytokines play important roles in mediating inflammation in autoimmunity. Several cytokines are elevated in serum and synovial fluid samples from children with Juvenile Idiopathic Arthritis (JIA). Soluble CD154 (sCD154) is elevated in other autoimmune disorders, but has not been characterized in JIA. Our objectives were to determine if sCD154 is elevated in JIA, and to examine correlations between sCD154 and other inflammatory cytokines.

**Methods:**

Serum from 77 children with JIA and 81 pediatric controls was analyzed for interleukin (IL)1β, IL2, IL4, IL5, IL6, IL8, IL10, IL12, IL13, sCD154, interferon-γ (IFNγ), soluble IL2 receptor (sIL2R), and tumor necrosis factor-α (TNFα), using the Luminex Multi-Analyte Profiling system. Differences in levels of cytokines between cases and controls were analyzed. Logistic regression was also performed.

**Results:**

sCD154 was significantly elevated in cases compared to controls (p < 0.0001). IL1β, IL5, IL6, IL8, IL13, IFNγ, sIL2R, and TNFα were also significantly elevated in JIA. Levels of sCD154 were highly correlated with IL1β, IL6, IL8, and TNFα (p < 0.0001). Logistic regression analysis suggested that IL6 (odds ratio (OR): 1.4, p < 0.0001), sCD154 (OR: 1.1, p < 0.0001), and TNFα (OR: 1.1, p < 0.005) were positively associated with JIA, while IL10 (OR: 0.5, p < 0.002) was protective. sCD154 was elevated in all JIA subtypes, with highest levels among more severe subtypes. IL1β, IL6, IL8, sIL2R and TNFα were also elevated in several JIA subtypes.

**Conclusion:**

Serum levels of sCD154, IL1β, IL6, IL8, sIL2R and TNFα are elevated in most JIA subtypes, suggesting a major role for sCD154, and these cytokines and cytokine receptors in the pathogenesis of JIA.

## Background

Juvenile idiopathic arthritis (JIA) is a heterogeneous group of arthropathies of unknown etiology. Both genetic and environmental factors are believed to play a role in susceptibility to JIA. One way to improve the understanding of the etiopathogenesis of JIA is to define better biological phenotypes of JIA. A common feature of the different subtypes of JIA is a tumor-like expansion of the inflamed synovial tissue, which is infiltrated by inflammatory cells including macrophages, plasma cells and lymphocytes. The production of inflammatory cytokines by these cells is thought to be instrumental in the development and perpetuation of the inflammatory arthritis. Several cytokines secreted by activated macrophages and lymphocytes such as interleukin (IL)1, IL6, and tumor necrosis factor-α (TNFα) have been implicated in the perpetuation of inflammatory response in JIA [1]. Other studies have implicated IL1β, IL6, IL8, soluble IL2-receptor (sIL2R), and TNFα in JIA [2-9].

CD40 is a member of the TNF-receptor family which is expressed on the surface of antigen presenting cells including B-cells, activated macrophages, dendritic cells and monocytes [10]. Signaling through CD40 induces antigen presenting cells to express different immune accessory molecules important in cell-cell interactions [11]. CD40 interacts with CD40 ligand (CD40L or CD154) which is predominantly expressed on activated CD4+ T-cells [12]. CD154 is transiently expressed on activated T-cells and plays a crucial role in B-cell function [13]. Activated T-cells expressing CD154 can also interact with CD40 on endothelial cells to induce production of inflammatory cytokines [14]. The transient expression of CD154 allows T-cells to stimulate selected CD40-bearing cells to participate in the immune response. Abnormal expression of CD154 has been reported in patients with systemic lupus erythematosus (SLE) and rheumatoid arthritis (RA) [15,16].

In addition to existing in a membrane bound form, CD154 can also be released as a soluble molecule (sCD154), which has been shown to have biological functions [17]. Increased levels of sCD154 have been demonstrated in patients with SLE, [18], RA, [19], inflammatory bowel diseases (IBD) [20], systemic sclerosis [21,22] and Sjogren's syndrome [23]. To our knowledge the concentrations and possible role of sCD154 has not been investigated in JIA. Our objectives were to examine the levels of multiple cytokines including sCD154 in children with JIA and healthy controls, and to determine the association of sCD154 with other inflammatory cytokines reported to be elevated in JIA. We also sought to determine if sCD154 levels differed between the different subtypes of JIA.

## Methods

Serum samples were obtained from 77 children (63% female) who fulfilled the International League of Associations for Rheumatology (ILAR) criteria for the diagnosis of JIA [24]. There were 10 patients each with systemic JIA and rheumatoid factor (RF)-positive polyarticular JIA, 14 with RF-negative polyarticular JIA, 20 with persistent oligoarticular JIA, 11 with extended oligoarticular JIA and with enthesitis related arthritis (ERA), and 1 with undifferentiated arthritis. The mean age of onset of JIA in the patients was 7.5 years. A total of 81 pediatric controls (52% female) were also studied with a mean age of 12 years (range 7–16 years). Controls were children obtained from an Associated Regional and University Pathologists (ARUP) institute of clinical and experimental pathology children's normal values study. The majority of the cases and controls were of Northern European ancestry. Serum was separated from peripheral blood soon after being drawn from subjects and aliquots were stored at -80°C until the time of cytokine determination. Subjects were enrolled after providing informed consent, under protocols approved by the Institutional Review Board of the University of Utah.

Information about disease variables at the time of enrollment was available on 72 children with JIA. Active disease was defined as having arthritis in at least 1 joint. Seven children did not have active disease. Variables collected included number of affected joints, ESR, and medications used to treat JIA at the time of enrollment (table 1). At the time of collection, 71% were on a non-steroidal anti inflammatory agent, 46% were on a disease modifying anti-rheumatic drug, 25% were on methotrexate, 19% were on an anti-TNF agent, and 12% were on corticosteroids.

**Table 1:** Disease activity and medication information of children with JIA

Variable	All JIA	Systemic	RF	poly	Oligo	Ext	ERA
Number	77	10	10	14	20	11	11
Onset age (years)	7.5	5.4	10	8.0	7.3	4.4	10.6
Median joints (n)	2.5	5	8	7	2	2.5	3.5
ESR (mm Hg)	23	49	26	17	18	28	11
Median number of medications	1	2	1.5	1	1	1	1
Proportion on NSAIDS %	71	50	80	61.5	89	44	90
Proportion on DMARDS %	46	60	70	61.5	16	22	50
Proportion on methotrexate %	25	30	60	46.2	0	0	30
Proportion on an anti-TNF agent%	19	40	10	30	5	22	20
Proportion on Corticosteroids %	12.5	50	10	23	0	0	0

NSAID: non steroidal anti-inflammatory drug, DMARD: disease modifying anti-rheumatic drug

### Multiplexed Cytokine Assay

The Luminex Multi-Analyte Profiling system (Luminex Corp, TX), is a flow cytometry based instrument that allows multiple analytes to be assayed simultaneously in a single sample [25]. The technology is based on the process of internally labeling 5.6 μm polystyrene microspheres with two fluorescent fluorophores. As the microsphere passes through the flow cell, it is interrogated by two lasers. One laser identifies the microsphere based on the ratio of the two fluorophores contained within the microsphere, while the other laser quantitates the amount of analyte bound to the microsphere by the intensity of reporter fluorescence. The surface of each microsphere contains multiple carboxyl groups that function as sites for covalent ligand attachment. The amount of analyte bound to the microspheres is determined by the median fluorescence intensity (MFI) of the reporter molecule, phycoerythrin, which is conjugated to a secondary or "detection" antibody.

The multiplexed cytokine assay we used was developed in the ARUP Institute for Clinical and Experimental Pathology, University of Utah using a standard sandwich capture format and has been described previously [26,27]. Briefly, monoclonal antibodies to human IL1β, IL2, IL4, IL5, IL6, IL8, IL10, IL12 p70, IL13, sCD154, IFNγ, sIL2Rα, and TNFα were used as capture antibodies and coupled to carboxylated Luminex microspheres using a two-step carbodiimide reaction [28]. A standard curve for each cytokine was made using known concentrations of recombinant human cytokine. The subject's sample (75 μl) was diluted 1:2 using a sample diluent and incubated for 10 minutes on an orbital plate shaker before the addition of the capture antibody coupled microspheres to allow for absorption of heterophile antibodies [27]. The cytokine standards, control samples, patient samples and microspheres were incubated for 1 hour at room temperature on an orbital plate shaker using 96-well filter bottom microtiter plate (Millipore Corporation, MA) to allow for subsequent washing by vacuum filtration. This was followed by the addition of 100 μl of a mixture of 13 different biotinylated secondary monoclonal antibodies to complete the sandwich capture assay. Following a second 30 minute incubation on the orbital plate shaker and washing, 100 μl of 5 μg/mL of streptavidin conjugated R-phycoerythrin (Moss Substrates, MD) was added to each well. After a 15-minute incubation and final wash, the microspheres were resuspended in 100 μl of PBST (10 mM phosphate buffered saline with 0.02% Tween 20), and the 96-well microplate was placed in a Luminex 100 instrument with an automated microtiter plate handler. The MFI of the unknown subject's sample was then converted into a pg/mL value based on the known cytokine concentrations of the standard curve using a 5-parameter regression formula.

### Statistical Analysis

Results are expressed as means, median and inter-quartile ranges. As most of the cytokine levels were not distributed normally, Wilcoxon two-sample tests were used to compare levels of cytokines between cases and controls. Correlations were determined using the Spearman's rank order correlation test. We also performed a stepwise logistic regression analysis to evaluate the effect of different cytokines on disease status. To make the ranges comparable, levels of sCD154 and sIL2R were divided by a factor of 10 for the regression analysis. Odds ratios (OR) and 95% confidence intervals were calculated. The Hosmer and Lemeshow statistic was calculated to assess the goodness-of-fit of the regression model. Goodness-of-fit statistics examine the difference between the observed frequency and expected frequency for groups of patients. A p value < 0.05 would suggest that the model is not well calibrated. Secondary analyses included comparison of cytokine levels between different JIA subtypes versus controls. We also compared the disease activity and treatment characteristics of children in the top tertile for sCD154 with those in the lower tertile. All comparisons were performed using SAS 9.1.

## Results

The serum levels of IL1β, IL5, IL6, IL8, IL13, sCD154, IFNγ, sIL2R, and TNFα were highly significantly elevated in cases compared to the controls (Table 2). Levels of IL2, IL4, IL10 and IL12 were not significantly different between cases and controls. The levels of IL2, IL4, IL5, and IFNγ were lower than 5 pg/ml in over 90% of subjects, while the levels of IL12 were lower than 5 pg/ml in 85% of subjects. The median levels of IL2, IL4, IL5, IL12, and IFNγ, were ~0 pg/ml among both cases and controls (Table 2). For this reason, these five cytokines were not included in subsequent analyses.

**Table 2:** Serum cytokine levels in healthy pediatric controls and in patients with JIA.

	**Controls** **N = 81**			**Cases** **N = 77**			**P value**
		
**Cytokine**	**Mean**	**Median**	**IQR**	**Mean**	**Median**	**IQR**	
**IL1β**	6.8	0.6	0–3.5	120.9	11	3.4–73	**<0.0001**
**IL2**	1.3	0	0–0	12.9	0	0–0	0.31
**IL4**	0.6	0	0–0.2	5	0	0–0.9	0.07
**IL5**	0.2	0	0–0	7.7	0.4	0–1.6	**<0.0001**
**IL6**	1.7	0.6	0–1.8	196.7	14.2	4.8–114	**<0.0001**
**IL8**	2.3	2	0–4.2	149.5	19.3	0.6–91.3	**<0.0001**
**IL10**	3.3	2.0	1.6–3.2	8.2	2.9	1.3–8.4	0.07
**IL12**	5.4	0	0–3.2	3.9	0	0–1.4	0.44
**IL13**	0.8	0	0–0.4	178.7	19	0–181.1	**< 0.0001**
**sCD154**	58.2	19	4.2–73.2	336.1	211.4	148.7–363.9	**< 0.0001**
**IFNγ**	0.3	0	0–0	4	0	0–0	**0.001**
**sIL2R**	796.8	785.1	611.8–962.9	1125	967.3	553.7–1436.9	**0.026**
**TNFα**	2.9	0	0–0	88.5	15	0–70.7	**<0.0001**

IQR, Interquartile range; IL, Interleukin; sCD154, soluble CD154; IFNγ, interferon-γ; sIL2R, soluble IL2 receptor; TNFα, tumor necrosis factor-α; All values median (range) are expressed in pg/ml.

### Correlation and regression analyses

When we performed correlation analyses, we found that sCD154 was highly positively correlated with the pro-inflammatory cytokines IL1β, IL6, IL8 and TNFα (p < 0.0001), and is also positively correlated with sIL2R and IL13 (p < 0.01) (Table 3). We also tested whether sCD154 levels had a relationship to age to determine if there were age related changes in our cohorts. There were no correlations between age at the time of collection, and levels of sCD154 or any of the other cytokines. When we examined correlations between disease activity variables and cytokine levels in cases, we found that ESR positively correlated with IL6 (p < 0.02), and number of joints (p < 0.02). The levels of TNFα correlated positively with the number of joints (p < 0.05) and number of medications (p < 0.003). There were no correlations between sCD154 levels and disease activity variables.

**Table 3:** Spearman Correlation Coefficients of the cytokines.

	IL1 **β**	IL6	IL8	IL10	IL13	sCD154	sIL2R	TNFα
IL1 **β**	1.00	0.65	0.54	0.46	0.37	0.55	0.28	0.63
		<.0001	<.0001	<.0001	<.0001	<.0001	0.0003	<.0001
IL6		1.00	0.59	0.46	0.30	0.47	0.31	0.63
			<.0001	<.0001	0.0001	<.0001	<.0001	<.0001
IL8			1.00	0.29	0.18	0.38	0.17	0.44
				0.0002	0.03	<.0001	0.0327	<.0001
IL10				1.00	0.27	0.20	0.46	0.35
					0.0006	0.0107	<.0001	<.0001
IL13					1.00	0.22	0.19	0.32
						0.0057	0.017	<.0001
sCD154						1.00	0.23	0.45
							0.0038	<.0001
sIL2R							1.00	0.29
								0.0002
TNFα								1.00

IL, Interleukin; sCD154, soluble CD154; IFNγ, interferon-γ; sIL2R, soluble IL2 receptor; TNFα, tumor necrosis factor-α; Spearmann correlation coefficients between various pairs of cytokines and p values are shown.

Since these cytokines were correlated with each other, we performed regression analyses. The Hosmer and Lemeshow goodness of-fit statistic was not-significant (p > 0.11) suggesting the model is well calibrated and fits agreeably with the data. The logistic regression procedure identified four cytokines with statistical significance: IL6 with an OR of 1.4 (1.19–1.67, p < 0.0001), IL10 with OR of 0.46 (0.30–0.69, p < 0.001), TNFα with an OR of 1.08 (1.02–1.13, p < 0.005) and sCD40 with OR of 1.14 (1.07 to 1.21. p < 0.0001).

### Comparisons of cytokine levels among JIA subtypes and controls

**Figure 1:**
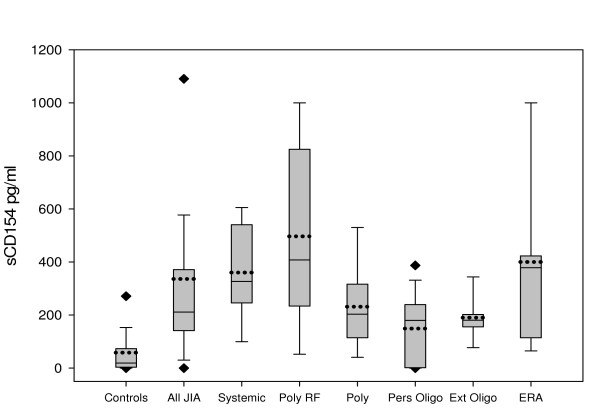
**sCD154 Levels among controls and children with different JIA subtypes**. Levels of sCD154 in different JIA subtypes. The boundaries of the boxes correspond to the 25^th ^and 75^th ^percentile. The solid horizontal line within the box denotes the median value and the dashed lines represent the mean values. The Whiskers above and below the boxes show the 90^th ^and 10^th ^percentiles respectively. Four subjects had levels greater than 1000 pg/ml.

We also compared the cytokine levels between patients with different JIA subtypes and healthy controls (Table 4). sCD154, IL1 β, IL6 and TNFα levels were remarkably elevated in all subtypes compared to controls albeit at different levels of significance (Table 4 and figure 1). Of note, sCD154 levels were most increased in children with RF+ polyarticular and systemic JIA as well as ERA than in the other types of disease. The highest levels of IL1 β was seen in patients with RF+ polyarticular JIA while IL6 was most elevated in patients with systemic and polyarticular JIA. IL8, IL10 and IL13 were significantly elevated in specific JIA subtypes compared to controls (Table 4). The highest levels of IL8 and IL13 were observed in children with polyarticular JIA. IL10 was significantly elevated in polyarticular as well as systemic JIA. Lastly, sIL2R was most associated with the more severe forms of JIA namely; systemic, RF+ and RF- polyarticular JIA.

**Table 4:** Cytokine levels in sera of healthy controls and in patients with different JIA subtypes.

**Cytokine**	**Controls** **N = 130**	**Systemic** **N = 10**	**Poly RF+** **N = 10**	**Poly RF-** **N = 14**	**Persistent oligo** **N = 20**	**Extended Oligo** **N = 11**	**ERA** **N = 11**
**IL1β**	0.6 (0–143)	6.8 (3–407)^§^	100.4 (0–1000)^§^	13.4 (0–1000)^§^	5.6 (0–289)^†^	29.7 (0–1000)^‡^	6.3 (1–656)^†^
**IL6**	0.6 (0–19)	34.2 (3–980)^§^	36 (1–1000)^§^	17 (0.9–1000)^§^	7.6 (1–1000)^§^	5.6 (1–1000)^§^	14.2 (2–99)^§^
**IL8**	2 (0–9)	14.2 (0–103)^‡^	62.6 (0–1000)^§^	63.3 (0–1000)^†^	12.4 (0–380)^§^	20.3 (0–1000)	0 (0–81)
**IL10**	2.0 (0–32)	6.7 (1–46)*	7.1 (1–40)*	3.9 (1–14)*	1.8 (0–42)	1.8 (0–10)	2.7 (1–76)
**IL13**	0.8 (0–15)	0 (0–427)	83.3 (0–1152)^‡^	48.4 (0–980)^†^	0 (0–1300)*	19 (0–396)*	19.4 (0–1367)*
**sCD154**	19 (0–599)	327 (89–609)^§^	408 (35–2382)^§^	203 (40–596)^§^	180 (0–390)*	180 (60–360)^§^	379 (57–2996)^§^
**sIL2R**	785 (180–1505)	1184 (703–3000)^‡^	1057 (174–2662)*	1355 (176–3000)*	501 (100–1762)	895 (273–2173)	822 (361–3000)
**TNFα**	0 (0–233)	22.8 (0–335)^§^	47.6 (0–564)^§^	24.3 (0–498)^§^	0 (0–191)*	66.8 (0–435)^§^	15 (0–1000)^§^

Poly RF+, rheumatoid factor positive polyarticular JIA; Poly RF-, rheumatoid factor negative polyarticular JIA; oligo, oligoarticular JIA; ERA, enthesitis related arthritis; IL, Interleukin; sCD154, soluble CD154; sIL2R, soluble IL2 receptor; TNFα, tumor necrosis factor-α;

All values median (range) are expressed in pg/ml. Values of sIL2R and sCD40L levels, as well as ranges of all cytokines have been rounded to the nearest number.

* Significant difference between controls and patients; p < 0.05.

‡ Significant difference between controls and patients; p < 0.01

† Significant difference between controls and patients; p < 0.001;

§Significant difference between controls and patients; p < 0.0001.

### Comparison of sCD154 levels with disease activity

When we compared children in the top tertile for sCD154 with those in the lower tertile, those in the upper tertile had a greater ESR (28 vs. 14) and greater median number of joints (4.5 vs. 2), but these differences were only marginally significant (p < 0.09). There were no statistically significant differences in the proportion of those on NSAIDs, disease modifying anti-rheumatic agents, methotrexate or anti-TNF agents between children in the top tertile for sCD154 and those in the lower tertile.

## Discussion

Utilizing a multiplex assay for cytokines and inflammatory markers, we characterized the profile of these biomarkers in a well defined cohort of JIA patients and controls. We observed striking elevations of a number of cytokines. Of special interest to us were the increased serum levels of sCD154 in children with different JIA subtypes. This elevation was most pronounced in children with systemic, RF-positive polyarticular and ERA subtypes, and modest among children with the oligoarticular subtypes. To our knowledge, ours is the first study to investigate sCD154 levels in JIA. Elevated levels of sCD154 have been found in other autoimmune disorders including SLE, RA, IBD, systemic sclerosis and Sjogren's syndrome [18-23]. Expression of CD40 on synovial monocytes, fibroblasts and dendritic cells in RA has been reported [29,30]. After stimulation in vitro, peripheral blood T-cells from patients with RA had increased and longer expression of CD154 compared to T-cells from controls [16]. Blocking CD40 on synovial fibroblasts from RA patients with soluble anti-CD40 antibodies prior to co-culture with synovial mononuclear cells resulted in decreased TNFα levels [16]. Moreover, blockade of CD154 with an antibody ameliorates collagen induced arthritis in a murine model of RA [31]. These observations suggest a major role for CD154 in the perpetuation of inflammation in RA.

The inflammation in synovial tissue of children with JIA is characterized by the presence of antigen presenting cells and activated T-cells, and is indistinguishable from the pathology observed in patients with RA [32]. Our results suggest that sCD154 might also play a role in the pathogenesis of most of the JIA subtypes. It is conceivable that the activated CD4 T-cells in JIA synovium may be the source of sCD154, although it is possible that other cells, such as activated platelets release sCD154. Cell-bound as well as sCD154 have been shown to activate endothelial cells *in vitro*, and increase production of leukemia inhibitory factor, IL6 and granulocyte-macrophage colony stimulating factors [14]. We speculate that sCD154 could possibly interact with cells bearing CD40, possibly enhancing antigen presentation, and production of other inflammatory mediators in JIA.

Our correlation analyses showed significant association between sCD154 and the pro-inflammatory markers IL6, IL1 β, IL8 and TNFα. It has been shown that sCD154 has the potential to activate endothelial cells *in vitro*, and increase production of IL6 [14]. Elevated levels of IL6 in JIA have been reported by several authors [2-7,33,34]. Indeed, levels of IL6 were strikingly higher among all JIA subtypes compared to controls in our study. IL6 was also significant in the logistic regression analysis suggesting that it likely plays a major role in concert with other cytokines in the pathogenesis of JIA. The increased levels of the pro-inflammatory cytokines IL1β and IL8 observed in our cohort have also been reported in different forms of JIA as well as RA [3-5,8,35]. In agreement with earlier findings, we observed that levels of TNFα were elevated in all subtypes except those with persistent oligoarticular JIA, in which fewer joint involvement and less systemic inflammation is observed [6,8]. Together these observations support a primary or secondary role for the inflammatory cytokines IL6, IL1β, IL8 and TNFα in different JIA subtypes.

The elevated levels of sIL2R and IL10 in the different subtypes of JIA are in agreement with previous studies [2,6,9] although with some subtle differences which may be attributable to the differences in the size of the cohorts. For example, although we observed increased levels of sIL2R in a JIA subtype equivalent to ERA in a study by Mangge et al [8], these concentrations were not significantly different from controls. Nevertheless, sIL2R levels tend to be highest in systemic or polyarticular JIA suggesting that it maybe a biomarker of JIA. Our logistic regression analysis suggests that IL10 might have a protective effect on disease, as might be anticipated by its inhibitory activity.

Some cytokines were barely detected in the serum in a majority of subjects detected in our study. For example, undetectable levels of IL2, and IFNγ have been reported by other investigators [34,36]. On the other hand, some of the results described here have not previously been reported by others. For instance some authors have reported finding no significant elevations in the levels of IL1β, IL8, or TNFα [2,4,8,33]. Differences in the results reported in this study and others could reflect heterogeneity of the different JIA cohorts. Other differences could be due to methodological issues, such as test specimen (serum versus plasma) and assay methodology. It is likely that some cytokines are more elevated in the synovial fluid and not in the serum, especially in children with persistently oligoarticular JIA.

Our study has several strengths. Our cohort of ~80 JIA patients and 80 pediatric controls is larger than most series. Our patients have been classified using the ILAR criteria, which results in more homogeneous subtypes compared to earlier studies. To our knowledge sCD154 has not been measured in JIA patients before. We also measured several other cytokines previously implicated in JIA. The multiplex assay used small volumes of serum to assay thirteen cytokines. This method has also been successfully used by de Jager et al[33], but sCD154 and sIL2R were not among the cytokines included in their study. They also found that IL6 differed between controls and patients. We have used an objective partially automated multianalyte assay which we have found to reliably measure cytokines and other analytes in a variety of disorders including immune deficiency [26], coronary artery disease [37] and acute rheumatic fever [38]. We believe the differences in cytokine profiles, as well as in the inflammatory marker sCD154, suggest that these might play a role in the pathogenesis of at least some subtypes of JIA.

The past decade has seen the emergence of biological therapeutic agents directed against several mediators of inflammation. Our findings of elevated TNFα is in agreement with the response seen with anti-TNF therapy in children with JIA. The elevated levels of IL1β and IL6 support the roles for biological agents targeting these cytokines as well. Finally, sCD154 could also be a potential target of biological therapy, although some studies in individuals with SLE raise the concern for pro-thrombotic effects, possibly due to effects of these agents on platelets and/or the endothelium [39]. If the importance of the CD40–CD154 pathway in JIA is confirmed, and safety concerns could be addressed, therapy directed at inhibiting this pathway deserves further exploration in JIA.

## Conclusion

We have demonstrated that sCD154 levels are elevated in sera from children with JIA. Furthermore, sCD154 levels correlated with several other pro-inflammatory cytokines, which are also elevated in children with JIA. The potential implications of sCD154 as a biomarker for treatment and monitoring patients is of particular appeal since sCD154 is a molecule associated with T cell activation and may play a role in the pathogenesis of JIA.

## Competing interests

The authors declare that they have no competing interests.

## Authors' contributions

SP conceived of the study, participated in the design and coordination of study, performed the statistical analysis, and wrote the manuscript, TBM Carried out the Luminex analyses, and helped to draft the manuscript, AET Participated in the design of the study, helped to coordinate the study, and to draft the manuscript, AW Participated in the design of the study, sample acquisition, and drafting of the manuscript, BC Enrolled subjects, involved in acquisition of samples and assisted with data collection, ASZ Participated in the design of the study, and helped to draft the manuscript, BMcN Participated in the design of the study, and helped to draft the manuscript, JFB Participated in the design of the study, and helped to draft the manuscript, HRH Senior author Participated in the design of the study, directed the Luminex analyses, and helped to draft the manuscript. All authors participated in the writing, read and approved the final manuscript.
